# A surgical recovery matrix to evaluate post-surgical recovery in mice using sham and myocardial infarction models of cardiac surgery as prototypes

**DOI:** 10.1371/journal.pone.0323317

**Published:** 2025-05-28

**Authors:** Eszter Pal, Brandon Shokoples, Saumya Naik, Kyle Hogue, Lorraine E. Chalifour

**Affiliations:** 1 Lady Davis Institute for Medical Research, Montréal, Québec, Canada; 2 Division of Clinical and Translational Research, Faculty of Medicine and Health Sciences, McGill University, Montréal, Québec, Canada; 3 Departments of Biology and Computer Science, McGill University, Montréal, Québec, Canada; University of Education, PAKISTAN

## Abstract

Examinations of biomarkers are useful in measuring overall health. Endpoints are critical to assess the threshold where the scientific aim of the study does not prevail over the wellbeing of experimental laboratory animal. However, parameters able to assess health and recovery after an acute post-surgical intervention are needed. To fill this gap, we combined a suite of qualitative and quantitative measurements and created a surgical recovery matrix (SRM) able to monitor and score rodent health and wellbeing after surgery. We established baseline values in healthy male and female retired breeder C57BL/6N mice as a control, no surgery (NoSx) cohort. To test if SRM scoring was useful in the immediate surgical period, we monitored changes in 18 parameters after sham (SH) and myocardial infarction (MI)-inducing surgeries over 3 days of recovery. The surgical manipulations and cardiac damage involved in MI-inducing surgery render its categorization as a major surgery. In contrast, SH surgery represents a minor surgery such that the surgical manipulations are identical to that of the MI surgery but without any manipulation/damage to the heart. Six hours after surgery, males and females showed deficits in nestlet integration, eye grooming, a hunched posture and rough coat appearance whereas greater body weight losses and impaired wound healing were recorded later in the observation period. Sex-specific differences were observed such that males showed a propensity to reduced mobility and lower surface body temperature whereas females had a reduced Body Condition Score. These parameters discriminated the very low scores detected in controls from the intermediate scores after SH surgery and the highest scores in mice severely debilitated by MI surgery. Further, we identified sex-specific and time-dependent changes such that the highest scores were detected in male mice after an MI versus SH surgery. We conclude that a combination of quantitative and qualitative parameters successfully evaluated mouse recovery and health after minor and major surgery.

## Introduction

To minimize the burden of animals in laboratory medicine, researchers monitor and reduce animal suffering via the formulation and consideration of ethical experimental, clinical and humane endpoints.” For example, Body Condition Scoring (BCS) is widely used as a clinical endpoint in the assessment of the physical health of multiple species, including the laboratory mouse [1]. It is effective because it is rapid to perform, needs no special equipment and is non-invasive.

Observational scoring systems have evaluated acute health during specific interventions such as infection. The Murine Sepsis Score [2], Modified Mouse Sepsis Score [3], Mouse Clinical Assessment Score for Sepsis [4], as well as a scoring system focused on activity in response to stimuli and eye appearance [5] showed that a group of physical signs could predict and presage death in mouse models of pneumonia and colon puncture sepsis. Common biomarkers included coat and eye appearance, behaviors such as eating, drinking and mobility, and responses to stimuli like touch or noise. Higher scores in the sepsis matrices aligned with increased circulating inflammatory cytokine levels, white blood cell and bacterial counts, and evidence of organ damage.

Laboratory animals may be subjected to interventions which have the potential to induce pain and discomfort. The Mouse Grimace Scale (MGS) is widely used to assess pain because it is simple, reproducible, and non-invasive [6,7]. Building on these data, the utility of tools to quantify changes in physical health (body weight fluctuation), behavior (burrowing and nest building) as well as pain management (MGS) were developed and tested in rodent surgery models [8]. Most studies have tested these matrices in mouse models of abdominal surgery such as laparotomy, cecal puncture ligation, biliary duct ligation, pancreatitis or models of abdominal inflammation and cancers [9–12].

To our knowledge, scoring systems to monitor or assess recovery from thoracic surgeries are lacking. To fill this gap, we report on our development of an observation-based matrix for murine models of cardiac and thoracic surgery. The Surgical Recovery Matrix (SRM) includes non-invasive physiological, behavioral, and post-surgical care measurements to produce an overall score indexed to the number of parameters monitored. Here, we describe the utility of the scoring matrix in retired breeder C57BL/6N male and female mice. We compare responses of mice after sham (SH) or myocardial infarction (MI)-inducing surgery with cohorts which did not undergo surgery (NoSx). The final score served as a risk level for compromised recovery and mortality, where a higher score indicated a higher risk of significant impairment. Overall, we found sex-specific and time-dependent changes in several parameters in SH and MI mice with the highest scores consistently found in male mice after MI.

## Methods

### Animal manipulation

All animal experiments were reviewed and approved by the Lady Davis Institute Animal Care Committee according to the guidelines of the Canadian Council on Animal Care. All laboratory staff successfully completed animal handling and surgical modules as stipulated by McGill University. Retired breeder C57BL/6N male and female mice, aged ~10–12 months, were purchased from Charles River Canada (Charles River, St. Constant, QC). A total of 30 male mice and 31 female mice were used in this study. Mice were provided with acidified tap water, housed in Allentown vented racks and polycarbonate cages with irradiated 1/4“corncob bedding (Teklad, Madison Wisconsin), and had a 12-hour dark/ light schedule with an average temperature of 24^o^C and humidity of 50%. Mice were supplied with a hut (non-recycled 100% wood pulp, BioServ, Flemington, NJ) and a nestlet (2” X 2”, pulped cotton fiber, Ancare, New York, NY). Upon arrival male mice were housed individually and female mice were housed in groups of 4.

### Diets and pre-surgery treatment

Mice consumed the house diet (HD, 2018 Teklad Irradiated Global 18% Protein Rodent Diet, 2918, Inotiv) during a 3-week acclimation. After acclimation, mice were maintained on the Total Western Diet (TWD, TD.220524, containing 16.8% protein, 54.4% carbohydrate and 16.7% fat, Inotiv). Both diets were commercially prepared, pelleted, and irradiated.

Mice remained on the TWD for the entire experimental period. Mice were randomly assigned to experimental groups and separated by sex. A no surgery (NoSx) group was used to determine normal baseline values for the parameters measured in the SRM. Two further groups were designed to determine the impact of surgery and events surrounding surgery. One group underwent sham (SH) surgery and the other underwent surgery to induce a myocardial infarction (MI).

### Surgery and treatments post-surgery

SH and MI surgeries were performed by an Animal Health Technician (Surgery and Phenotyping Core, Lady Davis Institute) who has performed these and other surgeries for over 14 years. Surgeries were completed within 20 minutes. On the day of surgery (D0), mice were anesthetized with 3% isoflurane 1.5 L/ min. oxygen, intubated, and injected subcutaneously with slow-release buprenorphine at 1mg/kg body weight, to supply analgesia for the following three days [13]. Ophthalmic ointment was applied to prevent eye dryness. Fur over the chest and abdomen area was removed with Nair^TM^, the torso washed and dried. For SH and MI surgery, a 1 cm incision was used to open the chest, the muscles over the ribs were separated, and a retractor inserted. The heart was visualized through an incision made between the third and fourth ribs. For MI surgery, the left anterior descending coronary artery, approximately 1 mm distal to the left atrial appendage, was ligated permanently using a 7–0 silk suture. Chest and skin wounds were closed with 6–0 and 7–0 silk sutures, respectively. Upon closure, one drop of prepared topical analgesia (1:1 mixture of lidocaine HCl (20 mg/ ml) and bupivacaine HCl, (5 mg/ ml)) was applied. Animals were injected subcutaneously with 1.0 ml warm saline, placed in their home cage and allowed to recover in a 35^o^C chamber for approximately1 hour before their return to general housing. The SH surgery protocol was identical to that of the MI protocol except that coronary artery ligation did not occur. After surgery, all mice were monitored twice daily by the Surgery and Phenotyping Care staff and once daily by the laboratory staff. Humane endpoints determined by the Animal Care staff, included the predetermined endpoints of piloerection, hunched posture and lack of mobility, with mice euthanized within one hour of notification. A total of 26 male and 27 female mice were used in this study. Four mice, all male and all after MI surgery, were found dead. Upon necropsy, they showed evidence of cardiac rupture as determined by a blood clot in the chest. Cardiac rupture is a sudden event.

### Surgical recovery matrix

Scoring took place before surgery, approximately 6 hours after surgery (D + 6h), and at approximately 9AM on post-surgery days 1, 2 and 3 (D + 1, D + 2 and D + 3, respectively). Mice were first observed in their home cage, placed on the cage top for close analyses and surface body temperature assessment and then moved to a 1-liter polypropylene beaker for movement analyses and fecal pellet collection. Once the assessment was completed, mice were placed back in their home cage. NoSx cohort observations were made at 9AM to correspond with the surgery cohorts.

A suite of physiological and behavioural parameters was prepared for monitoring, **Table 1**.

**Table 1:** Surgical recovery matrix.

Type	Parameter	Normal (0)	Concern (0.5)	Distress (1)
Surgical	Blood loss	None	<100μl	>100μl
	Post-anesthesia mobility	Active	Slow/ slightly active	Inactive
Physical	Eye appearance	Fully open	Half shut	Closed
	Skin color	Pink	Pale	White/blue
	Coat appearance	Flat & shiny	Up OR dull	Up AND dull
	Body condition score (BCS)	> 3	3	< 3
	Posture	Normal	Hunched (active)	Hunched (inactive)
	Mouse grimace score (MGS)	0	<1	>1
Physiological	% BW lost	< 5% lost	5–10% lost	>10% lost
	Suture intactness	Fully intact	<33% open	>33% open
	Wound healing	Pink/ healing	Red/ inflamed	Red/ infected
	Surface body temperature	> 31°C	30–31°C	< 30°C
	Food consumed/ day	> 3g	2 – 3g	<2g
Behavioral	Eye grooming	Cleaned	Partially cleaned	Not cleaned
	Nestlet integration	Fully integrated	Manipulated	Fully intact
	Mobility (home cage)	Active & curious	Slow & not curious	Inactive
	Mobility (novel)	Active & curious	Slow & not curious	Inactive
	Movement quality	Normal, fluid	Wobbling	Still & shaking

Table 1 **Surgical recovery matrix** Categorization of the values and signs of 18 parameters in the SRM considered as normal, of concern and an indicator of possible distress. A ready-to-use form is presented in S1 Appendix **Surgery Recovery Matrix Worksheet,** and a spreadsheet template in S2 Appendix**, Surgery Recovery Matrix Excel Worksheet.**

**Surgical parameters** included were related to events occurring during and shortly after the surgical intervention and were independently judged by the surgeon. Blood loss was scored as 0 if none, scored as 0.5 if < 100μl, and scored as 1.0 if > 100μl was lost. Mice were placed in their home cage in a 35^o^C incubator immediately after surgery. Post-anesthesia mobility was scored as 0 if actively mobile and awake within 15 min., scored as 0.5 if they required a further 30 min., and scored as 1.0 if greater than 1 hour of incubation time was required.

**Physical parameters** included visual inspection of external features including eye appearance, skin color, coat appearance, BCS, posture and MGS. Eye appearance was scored as 0 if open, scored as 0.5 if the eyes appeared half shut and scored as 1.0 if the eyes were closed. Skin color was measured at the chest and paws and was scored as 0 if normal if pink, scored as 0.5 if pale and scored as 1.0 if white or blue. Coat appearance was scored as 0 if shiny without piloerection, scored as 0.5 if piloerection was detected or dullness was detected and scored as 1.0 if piloerection and dullness were found. Posture considered whether the mouse was hunched and mobile. A score of 0 was noted if the mouse had normal posture, scored as 0.5 if hunched but mobile, and scored as 1.0 the mouse displayed hunched posture and was immobile. The BCS scoring [1] followed the guidelines of McGill University. A score of 0 indicated a BCS of ≥ 3.0, scored as 0.5 if the BCS was between 3.0 and 2.0 and scored as 1.0 if the BCS was < 2.0. Analyses of the MGS [6] was such that it was scored as 0 if all facial expressions remained not present, scored as 0.5 if the MGS was scored ≤ 1.0, and scored as 1.0 was given if the MGS was > 1.0.

**Physiological parameters** involved measurement of body weight, suture appearance, surface body temperature, fecal pellet number and weight, and food consumed. Body weight was quantified and scored as 0 if the loss was < 5% of their pre-surgery weight, 0.5 if between 5–10% of the pre-surgery weight was lost, and 1.0 if > 10% of the pre-surgery weight was lost. The incision site was judged on the intactness of the suture, appearance and color of the tissue. A suture was judged as 0 if intact along the length, scored as 0.5 if <one-third was open, and 1.0 if> one-third was open. Wound healing was scored as 0 if the skin was pink and healing, scored as 0.5 if somewhat red, and 1.0 if red and an infection was suspected. Surface body temperature was quantified using a hand-held infra-red thermometer (Kent Scientific, Corp., DT-811, CEM InfraRed Thermometer) held ~ 1 cm from the mouse perianal region. The surface body temperature was scored 0 if ≥ 31^0^C, scored as 0.5 if between 30 and 31^0^C and scored as 1.0 if less that 30^0^C. Mice were placed in a one-liter beaker for 15 minutes and fresh fecal pellets collected. The weight of the fecal pellets was measured and divided by the number of pellets to arrive at an average pellet weight. Fecal pellets produced from mice fed the TWD was scored as 0 if ≥ 10mg, 0.5 if between 5–10 mg and 1.0 if < 5 mg. Food consumption was diet and sex dependent. For males, TWD food consumption was scored as 0 if ≥ 3 g/ 24 hours were consumed, scored as 0.5 if between 1–3 g/ 24 hours were consumed, and scored as 1.0 if < 1.0 g/ 24 hours was consumed. For females, food consumption was scored as 0 if ≥ 2.8 g/ 24 hours were consumed, scored as 0.5 if between 1–2.8 g/ 24 hours were consumed, and scored as 1.0 if < 1.0 g/ 24 hours was consumed.

**Behavioural parameters** included eye grooming, nestlet integration, mobility in the home cage and novel environment, and the quality of the movement. Eye grooming was scored as 0 if the eye area was clean, scored as 0.5 if some mucous (dried or wet) was found, and scored as 1.0 if the eye area was not groomed and contained mucous (dried or wet). At the time of surgery, a new was placed at an area of the cage farthest from the pre-surgery nest. Nestlet integration was scored as 0 if the new nestlet was fully integrated into the mouse nest, scored 0.5 if the nestlet was moved closer to the mouse nest, torn or manipulated, and scored as 1.0 if the nestlet was not moved and remained untouched.

Mobility in the home cage was assessed when the cage lid was removed and scored as 0 if the mouse was active and appeared curious, scored as 0.5 if the mouse moved slowly, and scored as 1.0 if the mouse did not exit the hut or was inactive. Mobility in a novel environment was observed when placed in the one-liter bucket to collect fecal pellets. Mobility was scored as 0 if the mouse explored the area, moved freely and appeared curious, scored as 0.5 if the mouse moved slowly, showed hesitation and was not exploratory, and was scored as 1.0 if the mouse was immobile. Movement quality was scored as 0 if the mouse moved freely without hesitation, scored as 0.5 if movement was present but was shaky, and scored as 1.0 if the mouse was immobile and shaking.

### Echocardiography

On post-surgery day 3 (D + 3) and after the SRM was completed, mice were anesthetized with 3% isoflurane and 2L/ min. oxygen, placed supine on a heated platform, and ophthalmic ointment applied. Echocardiography was performed using a VEVO 3100 sonograph and MX550S transducer (VisualSonic, Toronto, ON.). Using VEVO Lab proprietary software, LV area and LV volume in systole and diastole and fractional area contracting (FAC) were calculated from EKV-gated long axis and short axis images, respectively [14,15]. FAC was defined as ((LV area in diastole minus the LV area in systole) divided by the LV area in diastole) X 100.

### Statistical analyses

Differences between groups were evaluated using student-t-tests for pairwise comparisons. Otherwise, comparisons were made using one-way ANOVA and Tukey-Kramer applied as a post-hoc test. A p-value of < 0.05 was considered significant. To assess whether an SRM value correlated with another SRM parameter or other data, Spearman’s correlation coefficient (r) and significance was calculated using IBM SPSS 22. A p-value of < 0.05 was considered significant. Heat map and Principal Component Analyses were performed using MATLAB version 9.14.0.2254940 (R2024a) update 2.

## Results

### Normal values and baseline SRM score

To establish normal values for SRM components, we evaluated a panel of parameters in 7 retired breeder male and 8 retired breeder female mice. **Table 1** lists the qualitative and quantitative parameters included in the SRM. Quantitative components of the SRM include body weight loss, surface body temperature, fecal pellet count and weight, and food consumed per day. Qualitative assessments monitored included their physical appearance, physiology and behaviour. Behavioral markers were the amount of food consumed/ day, eye grooming, nestlet integration, mobility, and the quality of the mobility in the home cage and in a novel environment.

**Fig 1:**
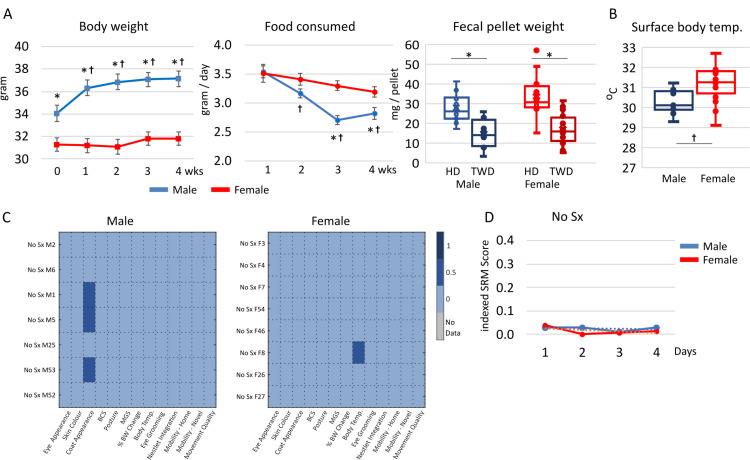
Baseline normal values. **A.** Physiological changes with time on the TWD. Retired breeder male, n = 7, and female, n = 8, C57BL/6N mice were acclimation to the house diet (HD) before the TWD. Body weight and food consumed were quantified weekly. Fecal pellets were collected, weighed and the average weight of each mouse calculated. Data are the mean ± SEM. A p-value of < 0.05 was considered significant and is indicated by an * in comparisons of HD and TWD and a † when males and females are compared. B. Surface body temperature at the perianal area was measured using a handheld infrared thermometer in mice fed the TWD. Data are the mean ± SEM. A p-value of < 0.05 was considered significant and is indicated by an † in comparisons of male and female mice. C. Thirteen parameters were measured, results scored as 0, 0.5 or 1.0 and a heat map for the male and female TWD-fed cohorts created. **D.** Parameters were measured each day for 4 days, scored, and the average of the indexed Surgical Recovery Matrix (SRM) plotted with time for each sex. The dotted lines indicate the trendlines.

To develop normal and baseline values for the quantitative parameters, the initial body weight, body weight change with time on the TWD, and the amount of food consumed were measured, **Fig 1A**. To complement the food intake data, we quantified the number of fecal pellets collectable within a 15 min. period and their average weight. We found that the number of fecal pellets produced was highly variable and therefore omitted this parameter in the SRM panel as an unreliable biomarker. We found that fecal pellets from mice fed the TWD weighed less and appeared drier and paler than those collected when the same mice were fed the HD, **Fig 1A**. Ultimately, initial and body weight changes with diet were sex-dependent and fecal pellet weight and appearance were found to be diet dependent. The surface body temperatures of male and female mice were measured to determine baseline values, **Fig 1B**. Using an infrared thermometer, measurement of the surface body temperature at the perianal region was selected as an easily accessible location unencumbered by the presence of fur and hence the need for depilation prior to temperature measurement. In this study, mice were placed on the cage top, the tail elevated, and the laser pointer of the infrared thermometer targeted on perianal area devoid of fur. We found a significantly higher surface body temperature in female versus male mice.

To develop a scoring system for quantitative parameters, the means and standard deviations were determined. A value of 0 was allocated to values greater than the mean, a value of 0.5 allocated when the measurement was between the mean and 1 SD below the mean, and a value of 1.0 was allocated when the measurement was greater than 1 SD value below the mean. For qualitative components of the SRM, such as skin color, a value of 0 was allocated when the attribute replicated that of normal mice, a value of 0.5 was allocated when attribute was present but did not mimic normal activity or the change was moderate, and a value of 1.0 was given when the attribute was absent or there was a marked change.

We collated scores for individual normal healthy mice and created a heat map, **Fig 1C**. The heat map indicates that the coat appearance of some healthy control retired breeder male mice displayed deficits in grooming. Additionally, one retired breeder female mice persistently had a surface body temperature which was lower than that of the other females. Next, we indexed the total score with the number of parameters assessed in each mouse to create a reference indexed SRM score. Using this system, a mouse with no impairments would have an overall SRM score of 0 and a mouse with the maximum deficits possible would have an overall SRM score of 1.0. Assessing the retired breeder males and females over a 4-day period revealed similar baseline indexed SRM profiles with scores hovering around 0.03 for male and female mice, **Fig 1D**.

### Cardiac structure/ function after surgery

**Fig 2:**
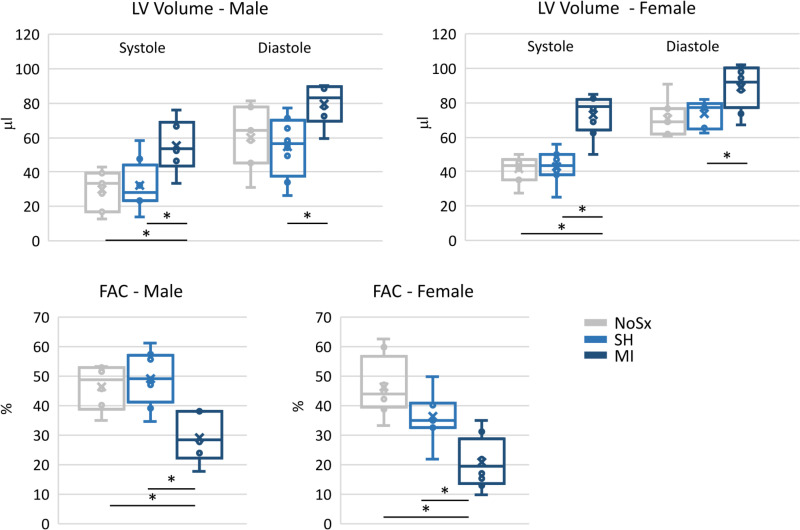
Echocardiography. Male and female mice were randomly assigned to no surgery (No Sx), sham (SH) or myocardial infarction (MI) surgery. Echocardiography was performed 3 days after surgery. Males; NoSx, n = 7; SH, n = 8; MI, n = 6; and Females; NoSx, n = 8; SH, n = 7; MI, n = 8 mice. The LV internal volume in systole and diastole, and LV fractional area change were calculated from EKV-gated acquisitions of the long axis view and short axis view, respectively. The box plots represent the median and minimum and maximum values for male and female mice. A p-value of < 0.05 was considered significant and is indicated by an *.

To identify parameters associated with minor and major surgery, we performed SH and MI surgery, respectively. To determine the effectiveness of the SH and MI surgeries, we evaluated left ventricle structure and function by echocardiography 3 days after surgery, **Fig 2**. As expected, SH surgery induced no change in cardiac structure or function in male or female mice. In contrast, expected and significant increases in the left ventricle volume and significant reductions in fractional area contracting were detected in male and female mice after MI surgery, indicating surgery-induced cardiac damage. These data validated the success of the surgeries.

### Impact of surgery on SRM parameters

**Fig 3:**
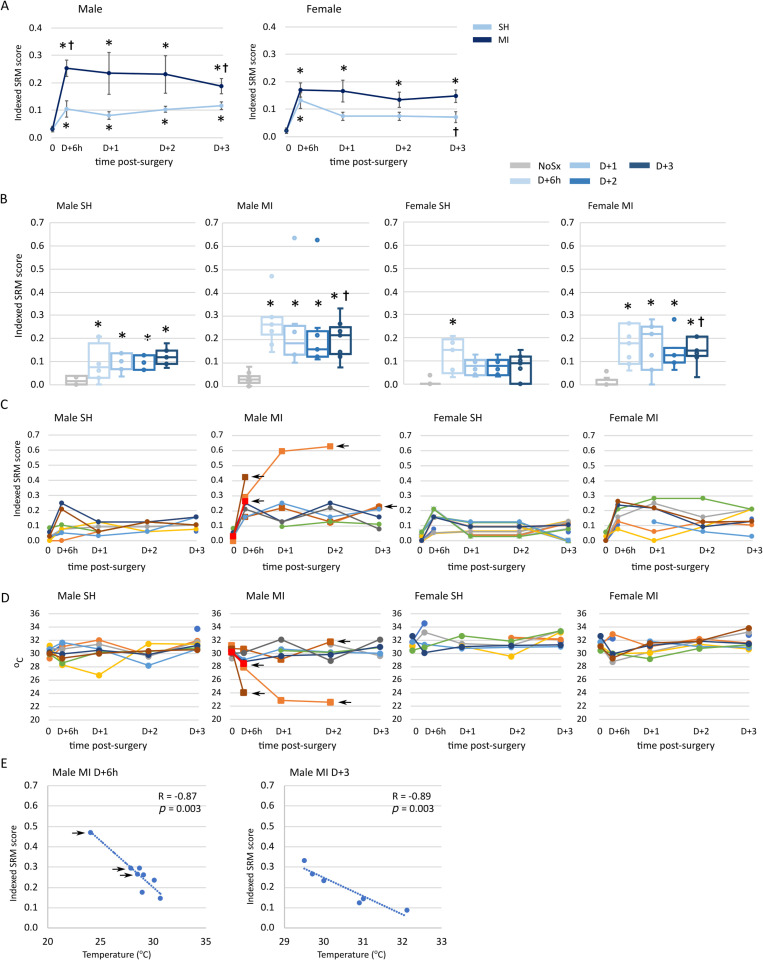
Surgical Recovery Matrix scores with time after surgery. Male and female mice were randomly assigned to no surgery (NoSx), sham (SH) or myocardial infarction (MI) surgery. Scoring was before surgery and with time after surgery. Males; NoSx, n = 7; SH, n = 7–8; MI, n = 6–8; and Females; NoSx, n = 8, SH, n = 5–7; MI, n = 7–8 mice per time point. A and B SH and MI indexed SRM scores with time after surgery in male and female mice. SRM scores were collected and indexed to the number of parameters assessed. Mice were examined before surgery (time 0), ~ 6 hours after surgery (D + 6h) and each day afterwards (D + 1, D + 2 and D + 3, respectively). Data are the mean ± SEM. A p-value of < 0.05 was considered significant and is indicated by an * in comparisons with time 0 values and a † when compared with the SH cohort. **C.** Indexed SRM scores for individual male and female mice before surgery and after SH or MI surgery. D. Surface body temperature for individual male and female mice before surgery and with time after SH or MI surgery. **E**. Correlation between surface body temperature and indexed SRM score for male mice 6 hours (D + 6h) and 3 days after surgery. Spearman R and *p* values are shown. Arrows indicate mice that died.

We measured the SRM parameters included in **Table 1** approximately 6 hours post-surgery (D + 6h) and daily thereafter until euthanasia on D + 3. Parameters were scored with reference to the normal values obtained in the NoSx cohorts of each sex, **Fig 1**. The total score was divided by the number of parameters measured to create an indexed SRM score for each mouse. The mean and SEM for male and female mice with time post-Sx are shown in **Fig 3A and 3B**. When compared with NoSx values, the indexed SRM scores of SH male mice were significantly increased at all times post-Sx but only at D + 6h in female mice. In males and females after MI surgery, indexed SRM scores were significantly higher when compared with NoSx scores at all time points. When indexed SRM scores were compared after SH or MI surgery, scores were higher in mice after MI surgery than those after SH surgery at D + 6h and D + 3 in males, and at D + 3 in females.

The LV internal volume in systole and diastole, and LV fractional area change were calculated from EKV-gated acquisitions of the long axis view and short axis view, respectively. The box plots represent the median and minimum and maximum values for male and female mice. A p-value of < 0.05 was considered significant and is indicated by an *.

To identify changes in individual mice, we plotted their indexed SRM scores with time post-Sx, **Fig 3C**. After SH surgery, peak indexed SRM scores were detected D + 6h after Sx in 2 male mice and 3 female mice with reduced scores detected over time. In male mice after MI surgery, we found a more variable response. Two male mice with sharp increases in indexed SRM values at 6 hours did not survive to the next day. Another male demonstrated an increase in indexed SRM score to 0.6 on D + 1 and D + 2 days and did not survive to D + 3. A fourth male survived until shortly before D + 3 without demonstrating an indexed SRM over 0.25. Of the remaining male mice, no mice died and indexed scores varied between 0.1 and 0.25. None of the female mice demonstrated such sharp increases in SRM score as in the males, and all female mice survived to D + 3.

We examined surface body temperature with time to determine if this parameter mirrored the patterns of the indexed SRM scoring, **Fig 3D**. Variations in body surface temperature were detected in SH male mice, particularly in an individual at D + 1 but were not found at later time points. In contrast, 2 of 3 male mice after an MI showed a reduced surface body temperature at D + 6h and did not survive to D + 1. The remaining mouse continued to show reduced temperature and succumbed before D + 3. Female mice after SH and MI surgeries demonstrated a wide variation in body temperature at D + 6h but had no obvious changes at later times.

To further examine the relationship between indexed SRM scoring and surface body temperature, Spearman’s correlation was performed. In mice after SH surgery, we found significant correlations of surface body temperature and indexed SRM only D + 6h after surgery, (Spearman R = - 0.534, *p* = 0.046) in males and after SH surgery only at D + 1 after surgery (Spearman R = - 0.809, *p *= .00089) in females. In male mice after MI surgery, we found significant correlation between surface body temperature and indexed SRM at D + 6h (Spearman R = - 0.886, *p* = 0.0034), D + 2 (Spearman R = - 0.881, *p* = 0.0086) and D + 3 (Spearman R = -0.949, *p* = 0.0039), **Fig 3E**. In contrast, no significant correlations were detected in female mice after MI surgery at any time point. We conclude that indexed SRM scoring identified male mice with a propensity to early death and that this was correlated with drops in body surface temperature.

**Fig 4:**
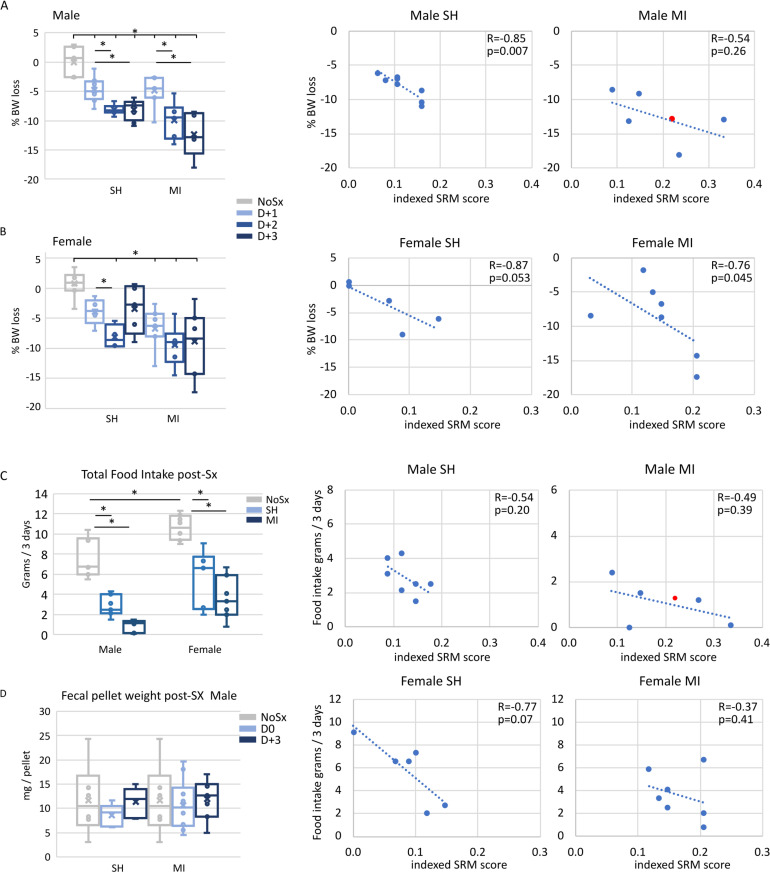
Body weight, food intake and fecal pellet weight after SH and MI surgery. A and B. Left: The percentage of body weight loss over each day of the 3-day recovery period was calculated for NoSx, SH and MI in male (A) and female (B) cohorts. Data are the mean ± SEM. A p-value of < 0.05 was considered significant and is indicated by an *. Right: Spearman correlation analyses examined the % body weight loss by D + 3 and the indexed SRM score on D + 3 for SH and MI cohorts. The red circle indicates the mouse which died the morning of D + 3. Shown are the trendlines, Spearman R^2^ values and *p* - values obtained. **C.** Left: Total food consumed of the over the 3-day recovery period was calculated for NoSx, SH and MI in male and female cohorts. Data are the mean ± SEM. A p-value of < 0.05 was considered significant and is indicated by an *. Right: Correlation analyses examined the % body weight loss by D + 3 and the indexed SRM score on D + 3 for SH and MI cohorts. The red circle indicates the mouse which died the morning of D + 3. Shown are the trendlines, Spearman R and *p* - values obtained. **D.** Fecal pellets were collected from male NoSx mice and after SH and MI surgery and the average pellet weight calculated, n = 5–8 mice/ group.

Body weight is a common biomarker for overall health. Male and female mice lost significant body weight after SH and MI surgery. Changes in percent body weight loss with time and correlations with indexed SRM values are shown, **Fig 4A and 4B**, respectively. After SH surgery, male and female body weight loss was significant on D + 1 and D + 2 after surgery but did not decrease further. In contrast, after MI surgery, male and female body weight was significantly reduced on all days post-Sx. Further, there was a significant correlation between total body weight lost and indexed SRM in male mice after SH (Spearman R = - 0.85, *p* = 0.007) but not after MI surgery (Spearman R = -0.54, *p* = 0.26). We noted was that the male MI mouse that died on D + 3 was midrange for body weight loss. Unlike the male mice, there was a significant correlation between total body weight lost and indexed SRM after MI (Spearman R = - 0.76, *p* = 0.045) but not after SH surgery (Spearman R = 0.87, *p* = 0.053) in female mice. Overall, the more invasive MI surgery resulted in greater loss of body weight in males and females than the less invasive SH surgery. However, the data suggest that body weight loss alone is not a strong indicator for a high SRM score or poor recovery.

Over the 3-day recovery period, food consumption was reduced in males and females after SH and MI surgeries with the greatest reductions found after MI surgery, **Fig 4C**. When compared, we detected no significant correlation between total body weight loss and total food consumption and no significant correlation between total body weight loss and the indexed SRM. Noted was that the male mouse that died at D + 3 post-MI surgery was midrange for food consumption. Further analyses of average fecal pellet weight found no differences in any cohort regardless of surgery or sex and no correlation with the indexed SRM scores, **Fig 4D**. These data suggest that body weight loss and changes in food consumption are significant after SH and MI surgeries but individually are not strong indicators of recovery after SH or MI surgery.

### Clustering of SRM parameters

**Fig 5:**
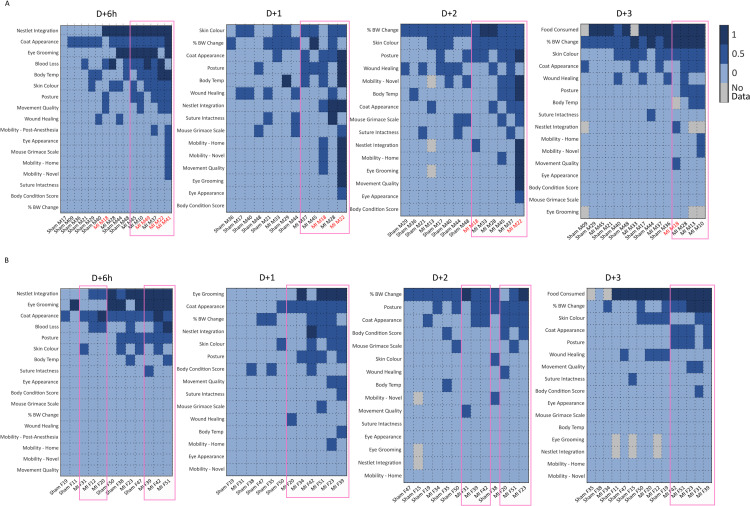
Changes in indexed SRM scores with time and post-surgery. SRM scores for each of the parameters indicated were measured on D + 6h, D + 1, D + 2 and D + 3 after SH or MI surgery as described. A heat map was prepared such that the highest scores for each parameter were grouped. The red box overlays indicate groupings of 3 or more MI mice. A and B. The scores for the indicated parameters for each mouse are shown for each time point in male (A) and female (B) SH and MI cohorts. The identification number of mice that died are in red font.

We summed the indexed SRM scores for 17 parameters at D + 6h, 15 parameters at D + 1 and D + 2, and 16 parameters at D + 3 post-Sx from each female or male mouse regardless of surgery. We then ranked the total parameter scores according to the individual mice to create heat maps for males, **Fig 5**, and females, **Fig 5B**. Each row was sorted from least (left) to greatest (right) revealing the lowest and highest scoring mice, and columns independently sorted from greatest (top) to least (bottom) revealing the lowest and highest scoring parameters, using the score sum of each parameter. The parameters with the highest scores were consistently found in male and female mice after MI surgery at all time points. Noted was that male mice which died during the night after D + 6h or on D + 2 displayed high scores in a greater number of parameters. While most male MI mice were clustered together at D + 6h, in the female mice, 2 separate groups of MI mice were identified at D + 6h and D + 2. From this, we conclude that while significant deficits in several parameters were shared between SH and MI mice, the indexed SRM score allowed for discrimination between mice after SH and MI surgery.

**Fig 6:**
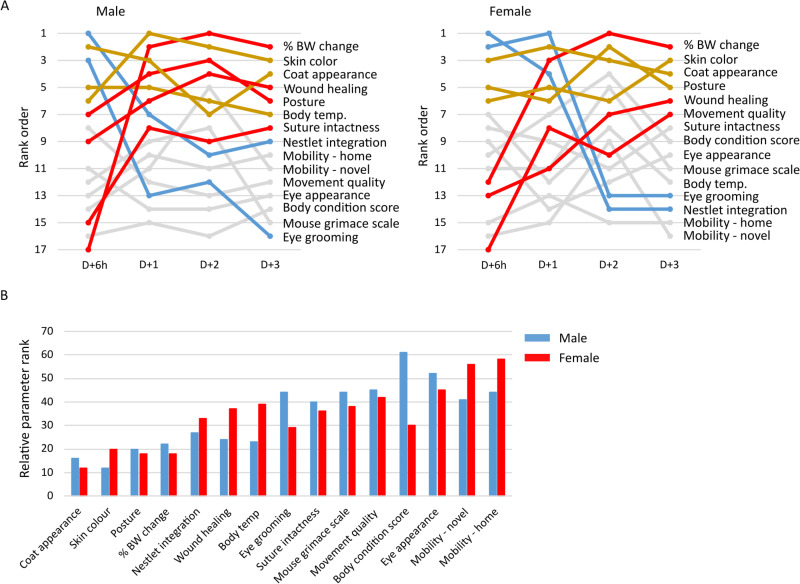
Time dependent changes in rank order of SRM parameters. Changes in the rank order of parameters revealed in Fig 5 were plotted with time regardless of surgery type. **A.** Changes in rank are color coded such that parameters that increase (red), reduce (blue), remain increased (gold) or form no pattern (gray) are indicated. **B.** The rank for each parameter for SH and MI cohorts in male and female was totaled and the values for males and females plotted.

To identify significant changes to parameter relevance with time, regardless of surgery type, we plotted the changes in ranking for each parameter based on score sums with time, **Fig 6A**. In this manner, a parameter that is highly affected and present in most mice would rank closer to 1. In contrast, a parameter showing minimal changes in only a few mice would rank closer to 17. In males and females, changes in skin color and coat appearance, along with posture in females, remained at a rank value of less than 5 at all time points. These data indicate that these markers, appearing first in the immediate surgical period, were not resolved as recovery progressed and remained highly relevant. Other parameters such as surface body temperature, nestlet integration and eye grooming in males, and eye grooming and nestlet integration in females, were affected soon after surgery but appeared resolved with time, thus dropping in rank. In contrast, larger numbers of male and female mice demonstrated increased percent body weight loss and deficits in wound healing at later time points post-Sx thereby increasing the rank order of these parameters over time. Additionally, in male mice, suture intactness and posture appeared to plateau on D + 1 and remain unresolved by D + 3. In female mice, movement quality was found to be impaired with time. Ultimately, we identified a number of SRM parameters that are consistently relevant throughout the recovery period in both sexes, while remaining SRM parameters show time- and sex-specific changes post-surgery.

To determine the most relevant SRM parameters to surgical recovery monitoring overall, we summed the scores for each parameter across all the time points regardless of surgery type and separated the values for males and females, **Fig 6B**. In this scheme, a low total indicates that the parameter was highly deficient in most mice and a relevant indication of impaired surgical recovery. On the other hand, a high total indicates that the parameter was unchanged by SH or MI surgery and/ or was relevant in few mice. It can be noted that the top 5 parameters were shared in importance in male and female mice. However, male mice demonstrated lower scores than female mice for some parameters suggesting some male-dependent deficits in wound healing, surface body temperature maintenance and mobility. In contrast, the scores of female mice were lower than males for eye grooming and BCS indicating loss of an ability to resolve these parameters. These data suggest that some aspects of recovery from surgery may be sex-specific and highlight certain parameters that are more relevant to males versus females in post-surgical monitoring.

**Fig 7:**
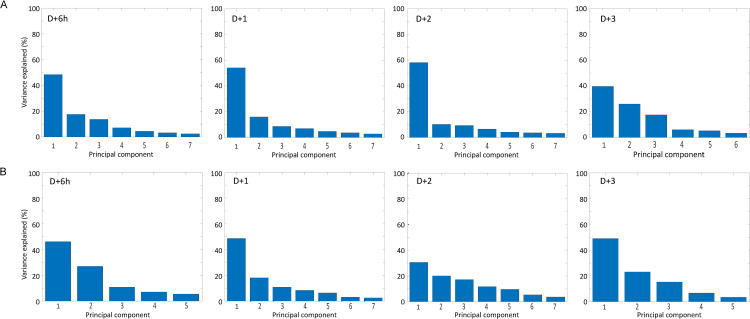
Principal component analyses. SRM scores for each of the parameters indicated were measured on D + 6h, D + 1, D + 2 and D + 3 after SH or MI surgery as described. PCA analyses were performed regardless of surgery type. A and B. Scree plots indicate the number of principal components formed in our analyses of male (A) and female (B) mice when SH and MI surgery were combined.

To further describe the dynamics of the SRM parameters and their contribution to the indexed SRM score we performed Principal Component Analyses, **Fig 7A and 7B** in males and females, respectively. Scree plots are valuable to identify the number of principal components (PCs)s and significance of parameter groupings to the overall score. In males, **Fig 7A**, and females, **Fig 7B**, a single Principal Component (PC1) forms much of the variance found on day D + 6h (48%), and D + 1 (54%), suggesting that a single group of parameters accounts for most of the SRM score. This predominance is maintained in males until D + 2 (58%) but is reduced on D + 3 (39%). Similarly, in females, a predominance of PC1 is present on D + 6h (46%), D + 1 (58%), and D + 3 (48%), whereas it is reduced on D + 2 (31%).

**Fig 8:**
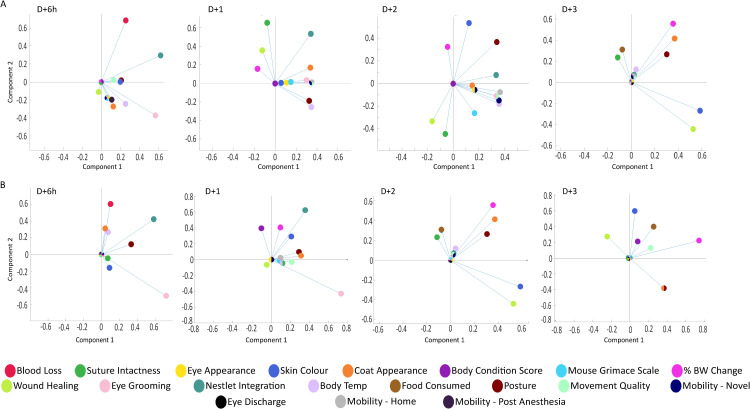
Loading plots of SRM parameters. A and B. Loading plots of the SRM variables in the Principal Components identified in Fig 7 in male (A) and female (B) mice when SH and MI surgery were combined. Each parameter is indicated by a colored circle. Lines indicate the distance from 0.

Loading plots bases on our Principal Component Analyses allow for the identification of the parameters contained within PC1 and PC2 in males (**Fig 8A**) and females (**Fig 8B**) with time. We observed that the parameters contained within PC1 changed with time and that these changes were sex dependent. In particular, nestlet integration and eye grooming were the main parameters identified in PC1 on D + 6h and D + 1 in males and females, and on D + 2 in males only. Whereas posture began to be an important indicator in PC1 and PC2 on D + 1 in males, it was a factor at all times in females. Coat appearance was noted as a consistently important marker in males and females after D + 1. Deficits in wound healing, skin color, and body weight loss were detected in PC1 and PC2 in males and females beginning on D + 3 and D + 2, respectively. Specifically detected in males were reduced surface body temperature and reduced mobility. In contrast, females demonstrated low BCS whereas this was unremarked in males. Ultimately, we conclude that clusters of SRM parameters were contained within PC1 and PC2 in mice recovering from SH or MI surgery.

## Discussion

It is increasingly recognized that multi-factor metrics are superior to single factor measurements when evaluating animal health. We deployed the SRM during the immediate post-surgical period and over a 3-day recovery period of retired breeder male and female C57BL/6N mice to test the utility of the SRM for evaluating and monitoring surgical recovery. Further, we sought to identify the parameters which would discriminate differences with surgery type comparing no surgery, sham surgery and MI surgery, with MI surgery being the most invasive and demonstrating a more involved recovery. We analyzed and compared data with time- and sex-dependent factors. Ultimately, we found time-dependent and sex-dependent changes in suites of parameters within the first 3 days of recovery distinguished between SH and MI surgeries. The highest scores indicating impaired recovery were found in male mice which died after an MI. Further, surviving male mice after the MI surgery had higher SRM scores than their female counterparts, while the lowest scores and thus least impaired recovery, were detected in female mice after SH surgery. Of the monitored SRM parameters, males and females at early times post-surgery showed deficits in nestlet integration, eye grooming, posture, and coat appearance, whereas at later timepoints greater body weight losses and impaired wound healing were shared. Specific to males was a propensity to reduced mobility and lower surface body temperature whereas females had reduced BCS. Our data show that lower surface body temperatures predicted early death in 3 of the 4 male MI mice that died. These data suggest that this measurement would be a useful forecaster of death in the post-surgical mouse as was demonstrated in rodent sepsis models [3,5]. Parameters aligned with successful recovery include female sex, early restoration of food intake and limited loss of body weight, maintenance of normal posture, mobility, and body temperature, timely suture healing and preservation of behaviors such as nestlet manipulation and grooming. Overall, the SRM revealed very low scores in NoSx controls, intermediate scores after SH surgery, and the highest scores in mice severely debilitated by MI surgery. Consequently, this scoring matric allowed for successful discrimination between the severity of the surgery and the corresponding severity of impaired in post-surgical recovery.

In this study, we selected a suite of parameters to evaluate murine physical health, appearance, and behavioral features to create the SRM. Our study aimed to develop and test a tool designed to assess recovery from cardiac/ thoracic surgery in mice, comparing no surgery, minor surgery and major surgery cohorts. We sought to employ observational methodologies that assessed physical and behavioural health in a non-invasive manner to achieve our goal of devising a tool able to quickly and easily determine health and welfare at the cage side. Other studies examining recovery from abdominal surgeries have instrumented the mice to assess heart rate, heart rate variability, core body temperature, and activity after a second intervention [10]. Additionally, others have added the quantification of circulating cytokines or corticosterone to their scoring matrices[10,16]. Although useful, a limitation to these methods includes the cost of the materials, time spent in recovery from the instrumentation, and longer time spent on data analyses. As described here, an SRM score sheet can be printed and completed or electronically completed while in the animal facility at the cage side. Later, the data can be entered into a spreadsheet and the indexed SRM score calculated within minutes. Our data demonstrate the ability of the SRM to discriminate between a less debilitating (SH) and more severely debilitating (MI) surgery.

To allow for cage-side monitoring, our quantitative measurements include body weight, food consumption require only a lab balance available in most procedure rooms and an easy calculation. In direct comparisons, infrared thermometer analyses versus implantable transponders were equivalent [17]. Based on this data, we opted for surface body temperature reading using an infrared thermometer to provide an instant readout and avoid the stress of anesthesia. Measurement at the perianal site avoided the need for restraint stresses. Previously, others have explored the sternum and perianal region as locations for surface temperature reading [18,19]. In SH and MI surgery, the torso is depilated at surgery offering a readily available location for testing. However, we found that temperature measurements fluctuated excessively while the mouse was restrained to view the torso compared with the steadier readings obtained at the perianal region in the mildly restrained mouse. Further, attempts to measure surface body temperature at the nape or chest resulted in highly variable temperatures possibly because of the need to move fur aside to allow assessment at the skin and the need to scruff the mice prior to assessment. Thus, measurement at the perianal site was chosen to avoid restraint stress.

Some of the SRM parameters, such as blood loss during the surgery, wound healing, suture intactness, post-anesthetic mobility and the MGS were included because they are relevant to events occurring during a surgery as well as to post-surgical wellbeing and recovery. Other measurements, such as BCS, body weight and temperature, posture, eye and coat appearance, skin color, nestlet integration and activity, are well recognized biomarkers of general animal health and are frequent components in matrices. Some matrices, such as the Murine Sepsis Score have summed the individual scores of each of their parameters to create a total score for each mouse [2,4] whereas others use the average of a set of components as a final score [3]. Like the Frailty Index [20,21], we chose to index the total SRM score for each mouse to the number of parameters monitored in that mouse. We reasoned that this would reduce the impact of any missing data to the final score. Overall, the SRM integrates assessments of physical, behavioural and surgical elements in a simple, easy-to-use matrix that outputs a final risk score evaluating post-surgical impairment.

To our knowledge, monitoring matrices in older animals after surgery are lacking. To establish normal, baseline values in our older mouse population and to determine sex-dependent norms, we monitored SRM parameters in healthy retired breeder and age-matched male and female C57BL/6N mice. We found some weeks of acclimation to a new diet were necessary to establish a steady body weight and observed sex-specific differences in food consumption. As expected, we found that retired breeder female mice had a significantly higher baseline surface temperatures than retired breeder males [22–24]. These data suggest that sex- and age-related physiology and accommodations to differences in diet must be factored into project planning including the determination of normal values and matrix thresholds prior to any application.

Reduced behavior-related measures such as eye grooming, nestlet integration, and burrowing were reported in some studies and linked to the anesthetic use and buprenorphine support necessary in a surgical application [8,16,25,26]. Our study found sex-differences in behaviors such as nestlet integration past D + 1, though no sex-dependent changes in the level of circulating buprenorphine in male and female mice are reported [13]. Specifically, male mice after MI but not SH surgery, and particularly those which later succumbed, showed a persistent deficit in nestlet integration despite equivalent doses or buprenorphine and anesthetic in all cohorts. These data suggest that the reduced nestlet integration in males is a result of impaired recovery induced by the more invasive surgery and unrelated to the identical buprenorphine and anesthetic support.

While food consumption was reduced in male and female mice after MI and SH surgery when compared with NoSx mice, we found no significant correlation between reduced food intake and greater body weight loss within the 3-day recovery period in any cohort. We speculate that the larger body weight of the mice at surgery (males averaged 37.9 ± 1.6 grams, females averaged 33.4 ± 1.2 grams) afforded sufficient adipose reserves to compensate for the reduced food intake early after surgery. Interestingly, we found no correlation between greater body weight loss and higher indexed SRM score in any cohort, and greater body weight loss was not detected in male mice which succumbed. Somewhat remarkably, we found no change in fecal pellet weight accompanied the reduced food intake. This suggests that fecal pellet weight is not a substitute for and does not reflect food consumption. Lastly, we detected no change in BCS in the short time frame of our study in heavier male mice but did find transient reductions in BCS in the lighter female cohorts.

The MGS is widely used to assess and monitor post-surgical stress in mice and rats after many types of surgeries [7]. Effective pain relief was found in many models of surgery, including MI surgery in 3-month-old female C57bl/6J mice [27]. However, most studies in mice have tested the MGS scoring systems in younger mice experiencing laparotomy [7,8] or in fracture models [26]. Our protocol used older retired breeder mice undergoing sham and MI surgery and the use of combined lidocaine for topical analgesia and slow-release buprenorphine at the dose predicted to provide effective analgesia for the 3 days of recovery [13,26]. Our buprenorphine formulation is designed to release peak drug amounts at 6 hours post-injection and maintain circulating buprenorphine levels above the effective threshold for 3 days. Despite our efforts, observations detected indications of pain as outlined by the MGS, in 1 of 16 male mice on D + 6h, 3 of 13 male mice on D + 1 and 4 of 14 male mice on D + 2 but none of 15 male mice on D + 3. On the other hand, fewer female post-surgical mice displayed features suggestive of pain. These data suggest potentially insufficient pain relief in this older age group, with a particular emphasis in the male cohort. Two remedial options are currently available: mice which demonstrate indicators of pain can receive a supplementary slow-release buprenorphine injection when appropriate [27] or extended-release buprenorphine with its higher recommended dose might be employed in older mice experiencing surgery.

Some parameters were omitted from the SRM because no significant or relevant changes were observed. For example, we saw no evidence of eye discharge or dehydration as assessed by tail color or skin tent retention and were not able to hear any vocalizations in any mouse. Other parameters, such as fecal pellet weight and fecal pellet appearance were affected by the changes in the diet but were unaffected by surgery in any mouse. Although consistently monitored, no mouse required extra fluids or extra time for recovery in the 35^o^C incubator on the day of surgery or any day after surgery. Lastly, parameters such as the number of fecal pellets produced, and time recorded for a 4-limb hang test were removed due to highly variability and unreliability in the SRM.

## Limitations

Our data and results are limited to the 3-day recovery period after surgery, and thus we cannot comment on whether the SRM would predict death or impaired recovery at later time points in the post-surgical period. Further, we did not explore other thoracic surgeries, or SH or MI surgery in younger mice to fully evaluate the impact of age. The number of male mice which did not survive the MI surgery to D + 3 was 4 out of 10 male or 60% survival. While the number of deaths is typical of the percentage survival we and others have obtained in SH and MI mouse models [15,28–31], it has limited our ability to unambiguously declare that the SRM can discriminate between mice expected to survive and those expected to require early euthanasia.

## Supporting information

S1 Appendix Surgery Recovery Matrix Worksheet. (DOCX)

S2 Appendix Surgery Recovery Matix Excel Worksheet. (XLSX)

S3 Appendix SRM_Raw mouse data. (XLSX)
