# Supplementary material for: A surgical recovery matrix to evaluate post-surgical recovery in mice using sham and myocardial infarction models of cardiac surgery as prototypes
Source: PLoS One. 2025 May 28;20(5):e0323317. doi: 10.1371/journal.pone.0323317 (PMC12118991; doi:10.1371/journal.pone.0323317)
Supplement: S1 Appendix — (DOCX) [file pone.0323317.s001.docx]

**Sx Date:**  **Analysis Date: Days Post-Sx:**

|  | **Parameter** | **Scoring** | **ID #** | **ID #** | **ID #** | **ID #** |
| --- | --- | --- | --- | --- | --- | --- |
| **Surgery** | **Blood loss** | None=0 \| 100μl=0.5 \| >100μl=1.0 |  |  |  |  |
|  | **Post-anesthesia mobility** | Active=0 \| Slow=0.5 \| Weak & unmoving=1.0 |  |  |  |  |
| **Appearance** | **Eye appearance** | Open=0 \| Half shut=0.5 \| Shut=1.0 |  |  |  |  |
|  | **Skin color** | Pink=0 \| Pale=0.5 \| White/blue=1.0 |  |  |  |  |
|  | **Coat appearance** | Shiny & flat \| Up OR dull=0.5 \| Up AND dull=1.0 |  |  |  |  |
|  | **Body condition score** | >3=0 \| 2=0.5 \| <2=1.0 |  |  |  |  |
|  | **Posture** | Not hunched=0 \| Hunched & mobile=0.5 \| Hunched & still=1.0 |  |  |  |  |
|  | **Mouse grimace scale** | Absent=0 \| Moderate=0.5 \| Severe=1.0 |  |  |  |  |
| **Physiology** | **Starting BW (gms)** | On day of Sx |  |  |  |  |
|  | **Current BW (gms)** | On day of analyses |  |  |  |  |
|  | **Change in BW** | Starting - current = change |  |  |  |  |
|  | **% BW lost score** | <5%=0 \| 5-10%=0.5 \|>10%=1.0 |  |  |  |  |
|  | **Suture intactness** | Intact=0 \| <33% open=0.5 \| >33% open=1.0 |  |  |  |  |
|  | **Wound healing** | Pink/healing=0 \| Red=0.5 \| Red & pussy=1.0 |  |  |  |  |
|  | **Body temperature** | >31°=0 \| 30°C-31°C=0.5 \| <30°C=1.0 |  |  |  |  |
|  | **Starting food weight (gms)** | On day of Sx |  |  |  |  |
|  | **Current food weight (gms)** | On day of analyses |  |  |  |  |
|  | **Food consumed (gms)** | Starting - current = change |  |  |  |  |
|  | **Food consumed (gms) / day** | Starting - current = change / # of days |  |  |  |  |
|  | **Food consumed score** | Normal=0 \| 2-3g=0.5 \| <2g=1.0 |  |  |  |  |
| **Behavior** | **Eye grooming** | Groomed=0 \| Partially=0.5 \| Not=1.0 | ­­­­ |  |  |  |
|  | **Nestlet integration** | Integrated=0 \| Moved/manipulated=0.5 \| Unmoved/untouched=1.0 |  |  |  |  |
|  | **Mobility (home cage)** | Active=0 \| Slow & not curious=0.5 \| Immobile=1.0 |  |  |  |  |
|  | **Mobility (novel)** | Active=0 \| Slow & not curious=0.5 \| Immobile=1.0 |  |  |  |  |
|  | **Movement quality** | Fluid/normal=0 \| Wobbly & mobile=0.5 \| Shaky & still=1.0 |  |  |  |  |
